# Suppression of STIM1 inhibits human glioblastoma cell proliferation and induces G0/G1 phase arrest

**DOI:** 10.1186/1756-9966-32-20

**Published:** 2013-04-11

**Authors:** Guilin Li, Zhenxing Zhang, Renzhi Wang, Wenbin Ma, Ying Yang, Junji Wei, Yanping Wei

**Affiliations:** 1 Department of Neurosurgery, Peking Union Medical College Hospital, Peking Union Medical College and Chinese Academy of Medical Sciences, No.1 Shuaifuyuan, Wangfujing, Dongcheng District, Beijing 100730, PR China; 2 Department of Neurosurgery, the First Hospital of Liaoning Medical College, People Street, Jinzhou, 121001, PR China

**Keywords:** Store-operated Ca^2+^ entry, Stromal interacting molecule 1, Proliferation, Cell cycle arrest, Human glioblastoma

## Abstract

**Background:**

Depletion of calcium (Ca^2+^) from the endoplasmic reticulum (ER) activates the ubiquitous store-operated Ca^2+^ entry (SOCE) pathway which sustains long-term Ca^2+^ signals and is critical for cellular functions. Stromal interacting molecule 1 (STIM1) serves a dual role as an ER Ca^2+^ sensor and activator of SOCE. Aberrant expression of STIM1 could be observed in several human cancer cells. However, the role of STIM1 in regulating tumorigenesis of human glioblastoma still remains unclear.

**Methods:**

Expression of STIM1 protein in a panel of human glioblastoma cell lines (U251, U87 and U373) in different transformation level were evaluated by Western blot method. STIM1 loss of function was performed on U251 cells, derived from grade IV astrocytomas-glioblastoma multiforme with a lentvirus-mediated short harpin RNA (shRNA) method. The biological impacts after knock down of STIM1 on glioblastoma cells were investigated in *vitro* and in *vivo*.

**Results:**

We discovered that STIM1 protein was expressed in U251, U87 and U373 cells, and especially higher in U251 cells. RNA interference efficiently downregulated the expression of STIM1 in U251 cells at both mRNA and protein levels. Specific downregulation of STIM1 inhibited U251 cell proliferation by inducing cell cycle arrest in G0/G1 phase through regulation of cell cycle-related genes, such as p21^Waf1/Cip1^_,_ cyclin D1 and cyclin-dependent kinase 4 (CDK4), and the antiproliferative effect of STIM1 silencing was also observed in U251 glioma xenograft tumor model.

**Conclusion:**

Our findings confirm STIM1 as a rational therapeutic target in human glioblastoma, and also indicate that lentivirus-mediated STIM1 silencing is a promising therapeutic strategy for human glioblastoma.

## Background

Human glioblastomas are the most common primary tumors of the central nervous system [1]. Despite recent development had been found in surgical resection, radiotherapy and chemotherapy, the prognosis has not changed significantly over the past two decades with a mean survival of less than twelve months from the time of diagnosis [2]. Therefore, new treatment strategies for glioblastomas is extremely needed. The increasing knowledge about genetic alterations that occur in glioblastomas has focused attention on development of targeted therapy which restore cell cycle or apoptosis defects in glioma cells. Therefore it could be an attractive alternative to conventional medicine [3-5].

Calcium (Ca^2+^) is a multifunctional messenger that control many cellular processes ranging from short-term responses such as muscle contraction and secretion to long-term regulation of cell growth and proliferation [6,7]. Store-operated Ca^2+^ entry (SOCE) is a major mechanism for Ca^2+^ entry across the cell membrane, which is stimulated in response to depletion of Ca^2+^ from intracellular Ca^2+^ stores (primarily the endoplasmic reticulum (ER)) and mediated via the activation of specific plasma membrane channels, termed as store-operated channels (SOCs) [8]. Stromal interacting molecule 1 (STIM1) is a highly conserved type-I membrane, ER-resident protein, containing a luminal EF-hand Ca^2+^-binding domain and several cytosolic protein-protein interaction domains, and serves a dual role as an ER Ca^2+^ sensor and activator of SOCE [9-11]. STIM1 initiates the process of store-operated Ca^2+^ influx by sensing the deletion of Ca^2+^ from the lumen of the ER store. It then migrates to the plasma membrane and forms aggregates at plasma membrane sites of Ca^2+^ entry and interacts either directly or in a complex with the plasma membrane-localized transmembrane protein Orai1 [9,10].

The role of STIM1 in regulating cancer progression remains controversial. In early investigations which were performed prior to the discovery of its role in Ca^2+^ signaling, STIM1 was described as a tumor suppressor for it causes growth arrest in human G401 rhabdoid tumor cells and human RD rhabdomyosarcoma cells [12,13]. However, subsequent studies revealed a potential role of STIM1 as an oncogene because it is up-regulated in several human cancers, such as breast cancer [14], glioblastoma [15,16] and cervical cancer [17]. Thus, more work needs to be done to fully determine the role of STIM1 in tumorigenesis which might vary in different tumor types. In the present study, we found that expression of STIM1 protein was higher in U251 and U87 glioblastoma multiforme (both Grade IV) lines than in U373 astrocytoma (Grade III), particularly higher in U251 cells [18]. Thus, we applied lentivirus-mediated small interfering RNA (siRNA) to suppress STIM1 expression and investigated the effects of STIM1 knock down on cell proliferation and cell cycle progression in U251 cells. Additionally, the proliferation-related markers (p21^Waf1/cip1^, Cyclin D1 and CDK4) were assessed in U251 cells with loss of function of STIM1. As a proliferation inhibitor, p21^Waf1/cip1^ was chosen because it is poised to play an important role in preventing tumor development. Cyclin D1-CDK4 complexes promote G1 phase progression through phosphorylation and inactivation of the retinoblastoma (Rb) gene product. Our results showed that specific downregulation of STIM1 inhibited human glioblastoma cell proliferation and induced G0/G1 phase cell cycle arrest by increasing expression of p21^Waf1/cip1^ and decreasing expression of Cyclin D1-CDK4. Therefore, STIM1 may serve as a therapeutic target for human glioblastoma.

## Methods

### Reagents and antibodies

Dulbecco’s modified Eagle’s medium (DMEM), fetal bovine serum (FBS), TRIzol^®^ Reagent and Lipofectamine™ 2000 were purchased from Invitrogen (Carlsbad, CA); 3-(4,5-dimethylthylthiazol-2yl-)-2,5-diphenyl tetrazolium bromide (MTT) (Dingguo biology, Shanghai, China); Dimethylsulfoxide (DMSO) (Shanghai Sibas Biotechnology Development Co., Ltd., China); 5-Bromo-2-deoxyuridine (BrdU) Cell Proliferation ELISA kit was purchased from Roche Applied Sciences (Indianapolis, IN); Giemsa was purchased from Chemicon International (Temecula, CA); Propidium Iodide (PI) was purchased from Sigma-Aldrich (St. Louis, MO); Bicinchoninic acid (BCA) Protein assay was purchased from HyClone-Pierce (South Logan, UT); M-MLV Reverse Transcription was purchased from Promega (Madison, WI); Oligo-dT was purchased from Sangon Biotech (Shanghai, China); SYBR green Master Mixture was purchased from Takara (Otsu, Japan); pFH-L vector and virion-packaging elements (packing plasmid mix) were obtained from Holybiol (Shanghai, China). Mouse anti-STIM1, mouse anti-GAPDH, p21^Waf1/Cip1^_,_ cyclin D1, cyclin-dependent kinase 4 (CDK4) and goat anti-mouse IgG were purchased from Santa Cruz biotechnology (Santa Cruz, CA), mouse anti-STIM2 was purchased from Abcam plc (Abcam, UK), mouse anti-Orai1 was purchased from Sigma biotechnology (Sigma- -Aldrich, US). All other chemicals were of analytical grade.

### Cell culture

Human kidney cell line HEK293,human glioblastoma cell lines, U251, U87 and U373, were all obtained from the American Type Culture Collection (ATCC, Manassas, VA) and cultured in DMEM containing 10% FBS, 100U/mL penicillin and 100 μg/mL streptomycin at 37°C in a humidified atmosphere containing 5% CO_2_.

### siRNA design and construction of recombinant lentiviral vector

Recombinant lentiviral vector was constructed as described previously [19]. The candidate sequence (5^′^-CCTGGATGATGTAGATCATAA-3^′^) in the *STIM1* cDNA sequence (GenBank accession number NM_003156) was selected for siRNA and blasted against the human genome database to eliminate cross-silence phenomenon with non-target genes. Scrambled siRNA (5^′^-TTCTCCGAACGTGTCACGT-3^′^) that does not target any genes was used as the negative control. Short hairpin RNA (shRNA) was constructed by annealing the synthetic DNA oligonucleotide primers, then naturally cooled to room temperature, and inserted between the *NheI* and *PacI* sites of lentiviral expression vector pFH-L which contains the green fluorescent protein (GFP) gene as a reporter with an internal CMV promoter. Clone identity was verified by sequencing.

Considering STIM1 CDS > 2 kb and inefficient expression of construct RESC lentiviral vector, another shRNA targeting the same gene STIM1 (NM_003156.3) was chosen to construct to get comparable results. The sense siRNA sequences were CGGCAGAAGCTGCAGCTGA and antisense siRNA sequences were TCAGCTGCAGCTTCTGCCG.

Recombinant lentiviral vector was produced by co-transfecting HEK293FT cells with lentiviral expression vector and packing plasmid mix using Lipofectamine™ 2000, according to the manufacturer’s instructions. Infectious lentiviral particles were harvested at 48 h post-transfection, centrifuged to get rid off cell debris, and then filtered through 0.45 μm cellulose acetate filters. The virus was concentrated by spinning at 4,000 g for 15 min following by a second spin (<1,000 g, 2 min). The titer of recombinant lentivirus was determined by serial dilution on 293 T cells.

### Recombinant lentivirus transfection in U251 cells

For lentivirus transduction, U251 cells were subcultured at 5 × 10^4^ cells/well into 6-well culture plates. After grown to 30% confluence, cells were transducted with STIM1-siRNA-expressing lentivirus (si-STIM1) or control-siRNA-expressing lentivirus (si-CTRL) at a multiplicity of infection (MOI) of 50. Cells were harvested at 72 h after infection and the transduction efficiency was evaluated by counting the percentage of GFP-positive cells.

### Quantitative real-time RT-PCR analysis

Total RNA from infected cells was isolated using TRIzol^^®^^ Reagent as recommended by the manufacturer. The quantity and purity of RNA were determined by UV absorbance spectroscopy. cDNA preparation was performed according to standard procedures using oligo-dT primer and M-MLV Reverse Transcriptase. Quantitative real-time PCR was performed by SYBR Green Master Mixture and analyzed on TAKARA TP800-Thermal Cycler Dice™ Real-Time System. The following primers were used for STIM1: 5^′^-AGCCTCAGCCATAGTCACAG-3^′^ (Forward), 5^′^-TTCCACATCCACATCACCATTG-3^′^ (Reverse); for p21^Waf1/Cip1^, 5^′^-GGGACAGCAGAGGAAGACC-3^′^ (Forward), 5′-GACTAAGGCAGAAGATGTAGAGC-3^′^ (Reverse); for cyclin D1, 5^′^-GGTGGCAAGAGTGTGGAG-3^′^ (Forward), 5^′^-CCTGGAAGTCAACGGTAGC-3^′^ (Reverse); for CDK4, 5^′^-GAGGCGACTGGAGGCTTTT-3^′^ (Forward), 5^′^-GGATGTGGCACAGACGTCC-3^′^ (Reverse). Housekeeping gene GAPDH was used as internal control and the primers are: 5^′^-AGGTCGGAGTCAACGGATTTG-3^′^ (Forward), 5^′^-GTGATGGCATGGACTGTGGT-3^′^ (Reverse). Thermal cycling conditions were subjected to 15 s at 95°C and 45 cycles of 5 s at 95°C and 30s at 60°C. Data was analyzed with TAKARA Thermal Dice Real Time System software Ver3.0. The gene expression change after lentivirus transduction was presented as relative expression (fold over the negative control or percentage of the negative control) after normalizing to GAPDH, and calculated using the 2^-ΔΔCt^ method as described previously [20].

### Western blot analysis

Lentivirus-transduced cells were washed twice with ice-cold PBS and suspended in a lysis buffer (2% Mercaptoethanol, 20% Glycerol, 4% SDS in 100 mM Tris-HCl buffer, pH 6.8). After 15 min of incubation on ice, cells were disrupted by ultrasound on ice. Total cell lysates were then centrifuged (12,000 g, 15 min, 4°C) and the supernatants were employed for further processing. The protein concentration was determined by BCA protein assay kit. Equal amount of proteins was loaded and separated by SDS-PAGE, and then transferred onto PVDF membrane (Schleicher&Schuell Co., Keene, NH) using an electro-blotting apparatus (Tanon, Shanghai, China). The membrane was blocked with 5% nonfat milk in TBST solution for 1 h at room temperature, and incubated overnight at 4°C with specific antibody to STIM1, p21^Waf1/Cip1^, STIM2, Orai1, cyclin D1 and CDK4 at the dilution 1:800, 1:1000, 1:800, 1:1000, 1:1500, and 1:1000, respectively. After three washes in TBST solution, the membrane was incubated with horseradish peroxidase-conjugated secondary antibody diluted with TBST solution at room temperature for 2 h. The signals of detected proteins were visualized on ECL plus Western blotting detection system (Amersham Biosciences, Inc., Piscataway NJ). GAPDH protein levels were used as a loading control.

### MTT cell viability assay and direct cell counting method

Cell viability was determined by a colorimetric MTT assay which described previously [21]. Briefly, lentivirus-transduced or TRPC entryway paralysed cells were seeded in 96-well plates at a density of 2 × 10^3^ cells/well. Ten microliters of MTT solution (5 mg/mL) was added into each well once daily for 5 days, and plates were incubated for 4 h at 37°C. After removal of the supernatant, 100 μL of DMSO was added to dissolve the crystals. The absorbance at 490 nm was detected with a microplate reader (Bio-Rad 680). Growth curve was performed according to the absorbance values (A) of 490 nm.

On the other hand, direct cell counting method was also used to cross-checking the results of MTT assay. Double target RNAi U251 cells were seeded in 96-well plates at a density of 1 × 10^4^ cells/well. After that, number of cells at 24 h and 48 h after seeding would be counted by blood cell counting plate. Besides, we count 3 wells for reduce error every time point. Growth curve was made according to the average number of cells in 3 wells.

### BrdU incorporation assay

Cell proliferation was also quantified by measuring BrdU incorporation during DNA synthesis using the BrdU Cell Proliferation ELISA kit. The experiment was performed according to the manufacturer’s protocol. Briefly, 10 μL/well of BrdU labeling solution was added to cells at 24 h and 72 h after culture. After overnight incubation, cells were fixed with 200 μL/well of fix solution for 30 min in the dark at room temperature, and then incubated with peroxidase-conjugated anti-BrdU antibody for 90 min in the dark at room temperature. A substrate solution was then added into each well, and absorbance was measured using microplate reader (Bio-Rad 680) at a wavelength of 450 nm with a reference wavelength of 630 nm. The number of proliferating cells is represented by the level of BrdU incorporation which directly correlates to the absorbance values. Growth rate (R) was calculated by the following equation:

$${R=(A}_{72h}{-A}_{24h}{)/A}_{24h}\times 100,$$

where A_72h_ and A_24h_ indicate the absorbance at 450 nm after 24 and 72 hours of incubation.

### Colony formation assay

Lentivirus-transduced glioblastoma cells (200 cells/ well) were seeded in 6-well plates. Culture medium was changed at regular time intervals. After 14 days of culture, adherent cells were washed twice with PBS, fixed with 4% paraformaldehyde for 30 min at room temperature. The colonies were stained with Giemsa solution for 15 min, then washed with water and air-dried. Cell colonies were counted using a light microscopy. The experiment was performed in triplicate.

### Cell cycle analysis

The effect of STIM1 on cell cycle distribution was determined by flow cytometry [22]. Briefly, lentivirus-transduced U251 cells (1 × 10^5^ cells/dish) were seeded at 6-cm dishes. Cells were harvested when they reach 80% confluence, and fixed at least 1 h with 70% ice-cold ethanol at 4°C. Cells were washed with PBS and resuspended in 1 mL of PBS containing 50 μg/mL PI and 100 μg/mL RNase A. After following incubation for 1 h in the dark at room temperature, cells were analyzed by flow cytometry using a FACSCalibur flow cytometer (Becton-Dickinson, San Jose, CA) at 24, 48 and 72 hrs after transduction. The fractions of cells in G0/G1, S, and G2/M phases were analyzed using dedicated software.

### Xenograft tumor model

The antitumor effects of siSTIM1 were evaluated *in viv*o using the U251 human glioma xenograft model in nude mice. All animal procedures were performed according to the guidelines of Peking Union Medical College Hospital, Peking Union Medical College and Chinese Academy of Medical Sciences. Briefly, U251 cells were infected with si-STIM1-expressing lentivirus or control-siRNA-expressing lentivirus at MOI of 50. When an apparent efficiency of 80%-90% GFP-positive cells was observed, cells were harvested and suspended at a density of 2 × 10^7^/mL DMEM (without serum), and 5 × 10^6^ cells were subcutaneously injected into the right dorsal flank of male BALB/c *nu/nu* athymic nude mice (SLAC Laboratory Animal Co. Ltd., Shanghai, China, 4-6 week-old) (n = 10 per group). The mice were housed and maintained under specific-pathogen free (SPF) condition. Tumor size was measured every 10 days using microcaliper, and tumor volume was calculated according to the following formula: tumor volume = (width^2^ × length)/2. The animals were sacrificed and tumors were excised after 30 days after injection. Tumor size was measured, and then the tumor was immediately frozen in liquid N2 for protein extraction.

### Protein extraction of xenograft tumors

Tumor tissue (approximate 5 mg) frozen in liquid N2 was manually homogenized on ice, lysed in lysis buffer supplemented with protease inhibitors, and then incubated on ice for 2 h. The supernatants were cleared by centrifugation (12,000 rpm, 20 min, 4°C). Protein extracts were used for assessing expression of STIM1 protein in the tumor samples by Western blot which described above.

### Statistical analysis

Data were expressed as the mean ± standard deviation (SD) of at least three independment experiments. The results were analyzed by Student’s *t*-test, and *P* < 0.05 was considered statistically significant.

## Ethical approval

All experimental research that is reported in the manuscript have been performed with the approval of Institutional Ethics Committee of Peking Union Medical College Hospital. Research carried out on humans be in compliance with the Helsinki Declaration, and all experimental research on animals follow internationally recognized guidelines.

## Results and discussion

### Expression of STIM1in human glioblastoma cell lines and HEK293 cell

**Figure 1:**
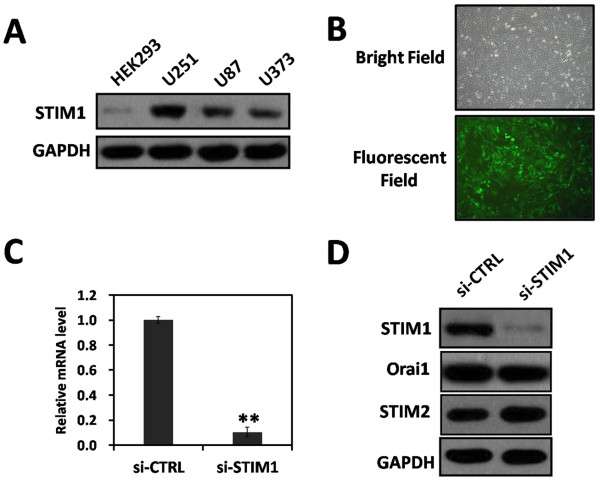
**Lentivirus-mediated siRNA inhibited STIM1 expression in U251 cells. **(**A**) Western blot assay: STIM1 protein is expressed in HEK293 cell and human glioblastoma cell lines of different transformation degree, as represented by U373 astrocytoma (WHO Grade III), U87 and U251 glioblastoma multiforme (WHO Grade IV) lines. (**B**) Transduction efficiency was estimated 72 hrs after transduction at MOI of 50. GFP expression in infected cells was observed under light microscope and fluorescence microscope. Light micrograph (top); Fluorescent micrograph (bottom) (×100). (**C**) Total RNA was extracted at 72 hrs after transduction and relative STIM1 mRNA expression was determined by quantitative real-time RT-PCR. GAPDH were used to standardize results. Data represent the mean ± S.D. of three independent experiments. **P < 0.01, compared with the si-CTRL group. (**D**) Total cellular proteins were extracted at 72 hrs after transduction and determined by Western blot analysis using antibodies against STIM1, Orai1, STIM2, with GAPDH as an internal control. Data represent one out of three separate experiments. si-CTRL: cells infected with control-siRNA-expressing lentivirus; si-STIM1: cells infected with si-STIM1.

To investigate the role of STIM1 in the malignant development of gliomas, we compared the expression levels of STIM1 protein in HEK293 cell and human glioblastomas cell lines in different transformation degree, as represented by U373 astrocytoma (WHO Grade III), U87 and U251 glioblastoma multiforme (WHO grade IV) lines by Western blot analysis. Of note, we chose HEK293 cell as a negative control of a non-tumor cell line for there was no normal glioma cell. As shown in Figure 1A, U251 cells, derived from a high-grade glioblastoma, showed higher expression of STIM1; therefore, U251 cells represent a reasonable cell culture system for experimental validations of data and were selected in the following loss of function experiments.

### Lentivirus-mediated siRNA targeting STIM1 inhibited STIM1 expression in U251 cells

To address the question of whether STIM1 could serve as a therapeutic target for human glioblastoma, we employed RNA interference (RNAi) method in an attempt to inhibit the expression of STIM1 in U251 cells. The efficiency of lentivirus transduction in U251 cells was examined by fluorescent microscopy, and more than 90% of the cells were infected with si-STIM1 at 72 hrs post-transduction at MOI of 50 as indicated by the expression of GFP (Figure 1B). To determine the knock down efficiency of STIM1, quantitative real-time RT-PCR and Western blot analysis were performed. As shown in Figure 1C, mRNA level of STIM1 in cells that infected with si-STIM1 was significantly decreased about 89.7% ± 3.8% compared with that in cells infected with control-siRNA-expressing lentivirus (si-CTRL) 72 hrs after transduction (**P < 0.01). Additionally, Western blot analysis was also performed 72 hrs after lentivirus transduction. Expression of STIM1 protein was significantly reduced in the si-STIM1 group in comparison to si-CTRL group while little effect on the expression of Orai1, and expression of STIM2 was compensatorily risen to a certain extent. (Figure 1D). Totally, these results indicated that lentivirus-mediated siRNA efficiently and specifically suppressed STIM1 expression in U251 cells.

### Suppression of STIM1 inhibited U251 cell proliferation

**Figure 2:**
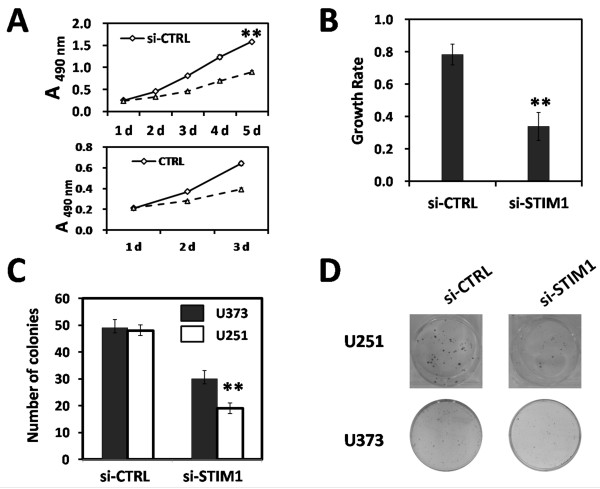
**Effect of STIM1 silencing on U251 cell proliferation. **(**A**) Cell proliferation of lentivirus-transduced and TRPC entryway paralysed U251 cell were measured by MTT assay once daily. Cell proliferation was expressed as the absorbance values. (**B**) DNA synthesis was measured by BrdU incorporation assay at 24 h and 72 h after transduction. Growth rate (R) was calculated by the following equation to reflect the role of STIM1 in DNA synthesis ability: R = (A_72h_ - A_24h_) / A_24h_ × 100 where A_72h_ and A_24h _indicate the absorbance at 450 nm after 24 and 72 hrs of incubation. (**C**), (**D**) Detection of cell proliferation by plate colony formation assay in U251 and U373cells. Representative photographs showing U251 and U373 cell colony in 6-well plate. U251 and U373 cells were seeded at 200 per well and allowed to form colonies. Cell colonies were scored visually and counted using a light microscopy. Data represent the mean ± S.D. of three independent experiments. **P < 0.01 compared with the si-CTRL group. si-CTRL: cells infected with control-siRNA-expressing lentivirus; si-STIM1: cells infected with si-STIM1.

The effect of down-regulation of STIM1 on proliferation of glioblastoma cells in *vitro* was assessed by MTT assay, BrdU incorporation assay and colony formation assay. Firstly, the amount of cell proliferation was determined using the MTT assay once daily for 5 days. As shown in Figure 2A, STIM1 silencing inhibited U251 cell proliferation in a time-dependent manner. When compared with the si-CTRL group, the cell number in si-STIM1 group was significantly reduced by 43.6% ± 3.5% (**P < 0.01) at 5 days post-transduction. Besides, after performed TRPC entryway paralysor SKF9636 in U251 cell, the malignant proliferation of U251 cell was observably slow down compared with CTRL group. The cell proliferation of U373 and U87 cells were shown in Additional file 1: Figure S1A and S1B. They had the same tendency compare with U251 cell. Cell proliferative activity was then assessed by BrdU incorporation into cellular DNA. Figure 2B shows a significant decrease the growth rate of U252 cells in si-STIM1 group (33.6% ± 5.8%) in comparison to si-CTRL group (78.1% ± 4.0%) (** *P* < 0.01).

At the same time, results of double target RNAi U251 cell viability detected by MTT assay and direct cell counting method were shown in Additional file 2: Figure S2A and S2B. They had the same tendency. And then, we detected expression levels of STIM1 protein by Western blot which could be seen in Additional file 2: Figure S2C.

Furthermore, the colony formation capacity in U251,U373 cells which infected with si-STIM1 or si-CTRL lentivirus was estimated at 14 days after transduction. As shown in Figure 2C and 2D, the number of U251 cell colonies in the si-STIM1 group (19) was reduced by 63.8% ± 4.6% (**P < 0.01) in comparison to the si-CTRL group (48) . The colony formation capacity in U373 cells was also shown in Figure 2C and 2D.

Collectively, these results showed that knock down of STIM1 by lentivirus-mediated siRNA could inhibit U251 cell proliferation in *vitro*.

### Suppression of STIM1 induced cell cycle arrest in G0/G1 phase and alterant expression levels of cell cycle-related genes in U251 cells

**Figure 3:**
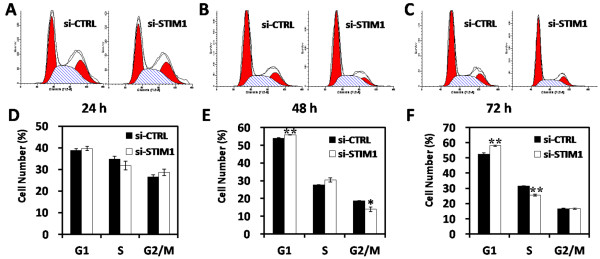
**Effect of downregulation of STIM1 on cell cycle progression in U251 cells. **Cell cycle distribution was performed by flow cytometric analysis. (**A**) Representative flow cytometric histograms at 24 hrs showing the distribution of cell cycle. (**B**) Representative flow cytometric histograms at 48 hrs showing the distribution of cell cycle. (**C**) Representative flow cytometric histograms at 72 hrs showing the distribution of cell cycle. (**D**) Knockdown of STIM1 by RNAi in U251 cells induced cell cycle arrest in G0/G1 phase at 24 hrs after transduction. (**E**) Knockdown of STIM1 by RNAi in U251 cells induced cell cycle arrest in G0/G1 phase at 48 hrs after transduction. (**F**) Knockdown of STIM1 by RNAi in U251 cells induced cell cycle arrest in G0/G1 phase at 72 hrs after transduction.

To further elucidate the growth suppression effect of si-STIM1 on U251 cells, we performed cell cycle distribution analysis by flow cytometry at 24, 48 and 72 hrs after transduction. As shown in Figure 3A, 3B and 3C, STIM1 knockdown induced cell cycle arrest in G0/G1 phase in U251 cells. When compared with the si-CTRL group, the percentage of G0/G1 phase in the si-STIM1 group was increased by 1.9% (*P < 0.05) at 48 hrs; what’s more, the percentage of G0/G1 phase in the si-STIM1 group was increased by 5.6% (*P < 0.05) at 72 hrs. The result demonstrate that STIM1 silencing may induce cell cycle arrest at G0/G1 phase and the effection of STIM1 on cell cycle does have time dependence.

**Figure 4:**
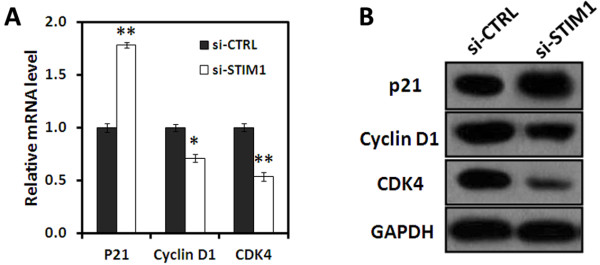
**mRNA and protein levels of cell cycle-related molecules. **(**A**) Total RNA was extracted at 72 hrs after transduction and mRNA expression of p21^Waf1/Cip1^_, _cyclin D1 and CDK4 was determined by quantitative real-time RT-PCR. GAPDH was used as an internal control. (**B**) Total cellular protein were extracted at 72 hrs after transduction and determined by Western blot analysis using antibodies against p21^Waf1/Cip1^_, _cyclin D1 and CDK4, and GAPDH as a loading control. Data represent the mean ± S.D. of three independent experiments. *P <0.05, **P < 0.01 compared with the si-CTRL group. si-CTRL: cells infected with control-siRNA-expressing lentivirus; si-STIM1: cells infected with si-STIM1.

We next performed real-time RT-PCR and Western blot analysis to assess the mRNA and protein levels of cell cycle-related molecules. After 72 hrs of transduction, RNAi-mediated STIM1 silencing induced upregulation of p21^waf1/cip1^ and downregulation of cyclin D1 and CDK4 simultaneously in U251 cells (Figure 4A and 4B). The results demonstrated that STIM1 may be involved in regulating the expression of cyclins-cyclin-dependent kinases (CDKs)-CDK inhibitors (CDKIs) in U251 cells.

### Lentivirus-mediated si-STIM1 inhibited tumor growth in *vivo*

**Figure 5:**
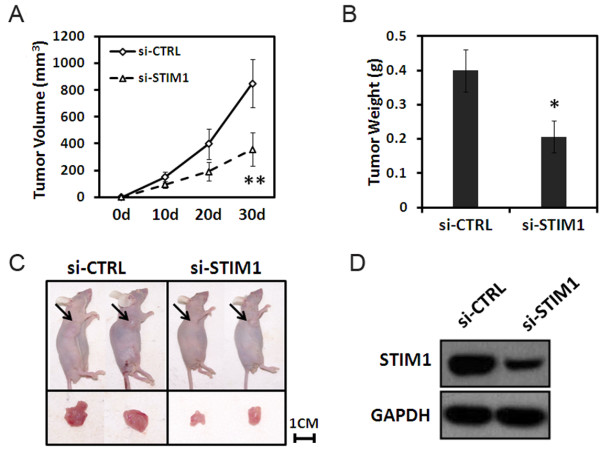
**Effect of STIM1 knockdown on tumorigenicity in nude mice. **U251 cells transduction with si-STIM1 or siCTRL were subcutaneously injected into the right dorsal flank of the nude mice as described in Materials and methods. Tumor volume was determined on Day 10, 20 and 30. At the end of the experiment, animals were sacrificed and tumors were excised for weight measurement and Western blot analysis. (**A**) Growth curve of tumor xenografts was assessed by serial microcaliper measurements. (**B**) Weight of tumor xenografts 30 days after inoculation. (**C**) Representative photographs of mice and tumors for each treatment. (**D**) Total protein from the resected tumors were prepared and used to determine the expression of STIM1 by Western blot analysis using antibodies against STIM1 and GAPDH. Data represent the mean ± S.D. of three independent experiments. *P <0.05, **P < 0.01 compared with the si-CTRL group. si-CTRL: cells infected with control-siRNA-expressing lentivirus; si-STIM1: cells infected with si-STIM1.

To determine whether STIM1 silencing could inhibit tumor development in *vivo*, lentivirus transduced U251 cells were subcutaneously injected into the right dorsal flank of the nude mice and tumor growth was evaluated. As shown in Figure 5A, the average growth rate of the ten si-STIM1 xenografts was reduced by 41.9% ± 5.7% (**P < 0.001, day 30) compare with control tumors (si-CTRL) as assessed by serial microcaliper measurements. si-STIM1 tumors that were resected on day 30 post-inoculation weighed 50% less than si-CTRL tumors (*P < 0.05) (Figure 5B). Representative photographs of mice in two groups (si-STIM1 and si-CTRL) and their transplanted tumors were shown in Figure 5C. Western blot analysis verified that STIM1 levels remained downregulation in the si-STIM1 transduced U251 xenografts in comparison to the control (Figure 5D). Thus, these results indicated that lentivirus-mediated gene silencing of STIM1 may be a promising therapeutic strategy for human glioblastoma.

## Discussion

SOCE, also known as capacitative Ca^2+^ entry, is thought to have an essential role in the regulation of contraction, cell proliferation, and apoptosis [23-25]. As a Ca^2+^ sensor in the ER, STIM1 is capable of triggering a cascade of reactions leading to SOCE activation [8], and involved in control of nontumorous cell proliferation [26-28]. Several studies have shown that STIM1 is overexpressed in human glioblastoma [15,16], but the molecular mechanism was not identified. Its role in regulating cancer cell proliferation and progression may be indirect and dependent on other Ca^2+^ entry proteins. Recent study by Liu et al. shows that calcium release-activated calcium (CRAC) channels regulate glioblastoma cell proliferation. Both Orai1 and STIM1 knockdown induced sustained proliferation inhibition in glioma C6 cells by using siRNA technology, being the effect of Orai1 silencing more prominent than that of STIM1 silencing [15]. Furthermore, Bomben and Sontheimer have recently shown that silencing the expression of TRPC1, a member of the family of TRPC channels also involved in SOCE, inhibits the proliferation of D54MG glioma cells and in *vivo* tumor growth [29].

In the present study, we found that STIM1 protein was expressed in human glioblastomas cell of different transformation degree, especially higher expressed in U251 cells that were derived from a high-grade glioblastoma; therefore, these phenomenon represent a reasonable cell culture system for STIM1 loss of function experiment. We employ lentivirus-mediated siRNA to suppress STIM1 expression in U251 cells. More than 90% of the cells were infected at MOI of 50 as indicated by the expression of GFP at 72 hrs post-transduction (Figure 1B). Both STIM1 mRNA and protein expression levels in U251 cells were downregulated (Figure 1C and 1D). Furthermore, knockdown of STIM1 inhibited U251 cell proliferation by inducing cell cycle arrest in G0/G1 phase in *vitro*, and this inhibition of proliferation would be in connection with damage of functional integrity of Ca^2+^ which induced by STIM1 knock-down (Figures 2 and 3). Through U251 xenograft model in nude mice, we found that STIM1 silencing also significantly affect tumor growth in *vivo* (Figure 4). Thus, these findings showed that STIM1 silencing resulted in changes in cell cycle progression and exhibited in *vivo* effects in tumorigenesis.

Deregulated cell cycle progression is one of the primary characteristics of cancer cells [30]. Cell cycle progression involves sequential activation of CDKs whose association with corresponding regulatory cyclins is necessary for their activation [31,32]. As a critical modulator of G1 to S transition, increased expression of Cyclin D1 in cancer cells resulted in an uncontrolled growth advantage. Cyclin D-CDK4/CDK6 and cyclin E-CDK2 complexes regulate cell cycle entry from G1 to S phase, phosphorylate and inactivate the retinoblastoma (Rb) protein. Upon phosphorylation, Rb dissociates from E2F family of transcription factors and allows for E2F-dependent transcription to occur [33]. As shown in Figure 3C and 3D, STIM1 silencing in U251 cells resulted in a marked decrease in the expression of cyclin D1 and CDK4. On the other hand, the CDKIs p21 ^waf1/cip1^ and p27 ^kip1^ regulate the progression of cells in the G0/G1 phase of the cell cycle and induction of these proteins causes a blockade of the G1 to S transition, thereby resulting in a G0/G1 phase arrest of the cell cycle [34]. The loss of CDKI in human cancers leads to uncontrolled cell proliferation which due to an increase in the levels of the CDK-cyclin complex [35]. In present study, STIM1 silencing caused a marked increase in expression of p21 ^waf1/cip1^ in U251 cells (Figure 3C and 3D). These observations suggest that STIM1 may play an important role in cell cycle progression of human glioblastoma by regulating the cyclins-CDKs-CDKIs expression.

The mechanisms linked to the inhibition of cell proliferation and tumor growth after STIM1 silencing were rather similar to our previous report which we show that RNAi-mediated silencing of the protein iASPP also results in G0/G1 cell cycle arrest in glioblastoma U251 cells, with concomitant changes in the expression of cyclin D1 and p21^wafl/cip1^[36]. However, subsequent study of the signaling pathway which regulates STIM1 function in glioblastoma still needs to be elucidated.

## Conclusions

In conclusion, we report that STIM1 is expressed in human glioma cell lines derived from a high-grade glioblastoma. RNAi-mediated gene silencing of STIM1 suppresses U251 cell growth both in *vitro* and in *vivo*, and blocks cell cycle progression at the G0/G1 phase. The anticancer effect of STIM1 silencing is likely mediated through the regulation of a large number of genes involved in cell cycle control, including p21^Waf1/Cip1^, cyclin D1 and CDK4. Thus, our findings illustrate the biological significance of STIM1 in tumorigenesis of glioma, and provide evidences that STIM1 may be a potential therapeutic target for human glioblastoma.

## Competing interests

The authors have no conflict of interests.

## Authors’ contributions

GL, ZZ and YW conceived, coordinated and designed the study, and contributed to the acquisition, analysis and interpretation of data and drafted the manuscript. RW, WM, YY, and JW performed the experiment and involved in drafting the article. YW accepts full responsibility for the work and/or the conduct of the study, had access to the data, and oversaw the decision to publish. All authors read and approved the final manuscript.

## Supplementary Material

Additional file 1: Figure S1 Effect of STIM1 silencing on U87 and U373 cell proliferation. (**A**) Cell proliferation of lentivirus-transduced U87 cell were measured by MTT assay once daily. (**B**) Cell proliferation of lentivirus-transduced U373 cell were measured by MTT assay once daily. Cell proliferation was expressed as the absorbance values. Click here for file

Additional file 2: Figure S2 Specific knockdown of STIM1 in U251 cells. Cell proliferation of double targets RNAi U251 cell were measured by MTT assay (**A**) and direct cell counting method (**B**) once daily. Cell proliferation was expressed as the absorbance values. (**C**) Western blot detecting for STIM1 in si-CTRL group and si-STIM1 group. Click here for file
