# Supplementary figures and images for: Suppression of STIM1 inhibits human glioblastoma cell proliferation and induces G0/G1 phase arrest
Source: J Exp Clin Cancer Res. 2013 Apr 11;32(1):20. doi: 10.1186/1756-9966-32-20 (PMC3639102; doi:10.1186/1756-9966-32-20)

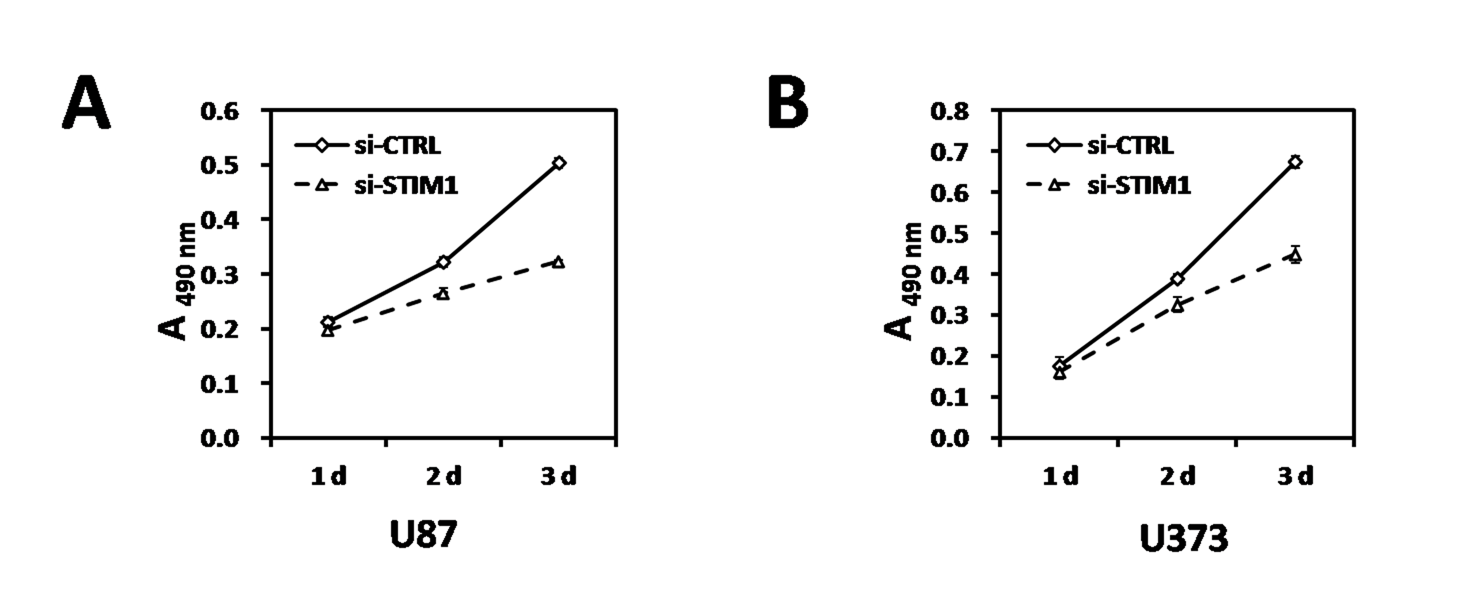

Supplement: Additional file 1: Figure S1 — Effect of STIM1 silencing on U87 and U373 cell proliferation. (A) Cell proliferation of lentivirus-transduced U87 cell were measured by MTT assay once daily. (B) Cell proliferation of lentivirus-transduced U373 cell were measured by MTT assay once daily. Cell proliferation was expressed as the absorbance values. [file 1756-9966-32-20-S1.tiff]

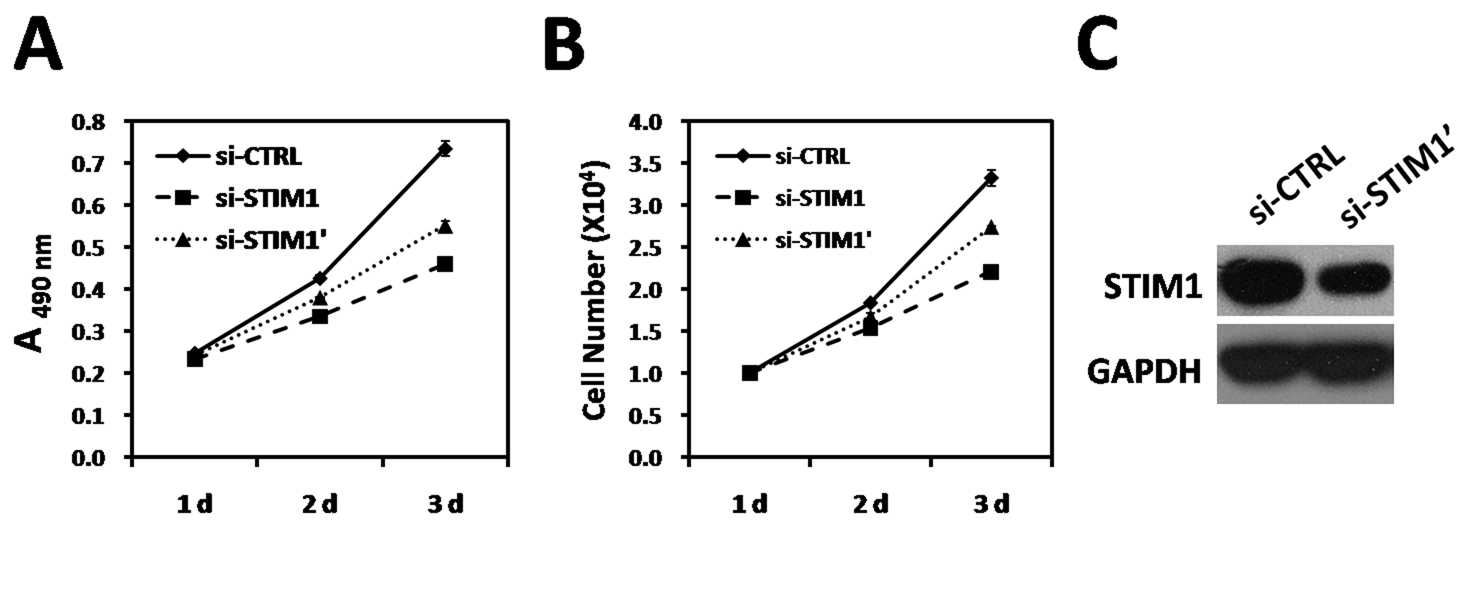

Supplement: Additional file 2: Figure S2 — Specific knockdown of STIM1 in U251 cells. Cell proliferation of double targets RNAi U251 cell were measured by MTT assay (A) and direct cell counting method (B) once daily. Cell proliferation was expressed as the absorbance values. (C) Western blot detecting for STIM1 in si-CTRL group and si-STIM1 group. [file 1756-9966-32-20-S2.tiff]
